# Identification of Novel Alzheimer’s Disease Loci Using Sex-Specific Family-Based Association Analysis of Whole-Genome Sequence Data

**DOI:** 10.1038/s41598-020-61883-6

**Published:** 2020-03-19

**Authors:** Dmitry Prokopenko, Julian Hecker, Rory Kirchner, Brad A. Chapman, Oliver Hoffman, Kristina Mullin, Winston Hide, Lars Bertram, Nan Laird, Dawn L. DeMeo, Christoph Lange, Rudolph E. Tanzi

**Affiliations:** 1 Genetics and Aging Unit and McCance Center for Brain Health, Department of Neurology, Massachusetts General Hospital, Boston, MA USA; 2 Harvard Medical School, Boston, MA USA; 3 Department of Biostatistics, Harvard T.H. Chan School of Public Health, Boston, MA USA; 4 Channing Division of Network Medicine, Brigham and Women’s Hospital, Boston, MA USA; 5 Department of Clinical Pathology, University of Melbourne, Victoria, 3000 Melbourne, Australia; 6 Department of Neuroscience, Sheffield Institute for Translational Neurosciences, University of Sheffield, Sheffield, UK; 7 Department of Pathology, Beth Israel Deaconess Medical Center, 330 Brookline Avenue, Boston, MA US; 8 Lübeck Interdisciplinary Platform for Genome Analytics, Institutes of Neurogenetics and Cardiogenetics, University of Lübeck, Lübeck, Germany; 9 Department of Psychology, University of Oslo, Oslo, Norway; 10 Division of Pulmonary and Critical Care Medicine, Brigham and Women’s Hospital, Boston, MA USA

**Keywords:** Quality control, Genome-wide association studies, DNA sequencing, Alzheimer's disease

## Abstract

With the advent of whole genome-sequencing (WGS) studies, family-based designs enable sex-specific analysis approaches that can be applied to only affected individuals; tests using family-based designs are attractive because they are completely robust against the effects of population substructure. These advantages make family-based association tests (FBATs) that use siblings as well as parents especially suited for the analysis of late-onset diseases such as Alzheimer’s Disease (AD). However, the application of FBATs to assess sex-specific effects can require additional filtering steps, as sensitivity to sequencing errors is amplified in this type of analysis. Here, we illustrate the implementation of robust analysis approaches and additional filtering steps that can minimize the chances of false positive-findings due to sex-specific sequencing errors. We apply this approach to two family-based AD datasets and identify four novel loci (*GRID1*, *RIOK3*, *MCPH1*, *ZBTB7C*) showing sex-specific association with AD risk. Following stringent quality control filtering, the strongest candidate is *ZBTB7C (P*_inter_ = 1.83 × 10^−7^), in which the minor allele of rs1944572 confers increased risk for AD in females and protection in males. *ZBTB7C* encodes the Zinc Finger and BTB Domain Containing 7C, a transcriptional repressor of membrane metalloproteases (MMP). Members of this MMP family were implicated in AD neuropathology.

## Introduction

Alzheimer’s disease (AD) is the most common form of dementia worldwide, with a substantial burden for not only patients, but their families, society and the healthcare system. The impact of the disease is expected to increase further by 2050, with a projected 13.9 million Americans to develop AD or related dementias^1^. Like most complex diseases, AD is caused by a mixture of genetic and environmental factors. Early-onset familial AD (monogenic) is caused by rare fully penetrant mutations in *APP*, *PSEN1*, *PSEN2* genes^2^. The more prevalent form, late-onset (sporadic) AD, is caused by a complex polygenic architecture, including large-effect variants in the *APOE* gene^3^. Environmental and lifestyle factors also affect the prevalence of the disease, however this domain is less well elucidated to date. Although one of the strongest predictors for AD is age, there are several other risk factors, including race^4^, high blood pressure^5^, brain trauma^6^ and sex^7–10^.

Not only are women at twofold greater risk than men, the progression of the disease and neurodegeneration is more rapid among women versus men^11,12^. In contrast, men with AD have higher mortality, as compared to women^12,13^. Interactions between sex and *APOE* ε4 have been previously reported. For instance, Altmann *et al*. showed that women have greater AD risk in the presence of *APOE* ε4, and this *APOE*-related risk in women may be associated with tau pathology^14^. Another study showed opposite directions on cognition among male *APOE* ε4 carriers versus female *APOE* ε4 carriers during intranasal insulin treatment^15^. A few studies have assessed other genes or performed a systematic gene-by-sex genetic analysis. For example, female- or male-specific effects have been reported in *ACE*^16^, *BDNF*^17^ and *RELN*^18^ genes. Large-scale meta-analyses of genotyped data have largely focused on the AD affection status itself, rather than on sex-specific AD effects^19–21^. This may be attributed to the fact that understanding and modelling gene-by-environment interactions still remain major challenges in the field, due to lack of power given current analytic methods.

FBATs have been recognized to be robust to population structure and to have the advantage of flexible model building based on solely Mendelian transmissions^22^. This feature of FBATs becomes particularly important when statistical inference is made for an environment interaction effect in the context of WGS-based data, where most of the variants are rare. Adjustment approaches that are based on common variant data might not capture the population structure of the rare variant information. Furthermore, FBATs require only minimal assumptions in terms of modelling the phenotypes^22^. The correct specification of the phenotypic model increases the power of the FBAT, but a misspecification does not affect the validity of its test results, i.e. type-1 error. Several extensions of FBATs have been proposed for gene-environment interaction analyses^23–28^. While some of these do not scale well with WGS data and require several statistical assumptions, others are better suited for such analysis settings.

However, in the context of rare-variant data, family-based studies face one major hurdle: they are sensitive to genotyping/sequencing errors. In the context of sex-specific analyses, this issue is further aggravated as many genetic regions show sequence homology with the X-chromosome^29^. This can lead to differential genotyping error rates for females and males due to different X chromosome dosage. Ignoring the impact of such sex-specific genotyping/sequencing errors can lead to substantially inflated type-1 errors^29^.

Here, we sought to model sex-specific genetic effects within the traditional FBAT framework to analyze two WGS-based AD family datasets for sex-specific genetic associations. We discuss several approaches to test for locus-by-sex interactions in family-based designs where the analysis is restricted to affected individuals only. It is important to note that implementation of an affected-only approach in a population-based design is not straightforward, as the inclusion of covariates to adjust for population substructure is non-trivial^30^.

While affected-only analysis approaches can be implemented in the FBAT-framework, e.g. in application to AD-WGS datasets, they can be sensitive to sex-specific genotyping errors that are not filtered out by standard QC-pipelines. To minimize such effects, we also discuss the implementation of additional QC filters in this setting.

Our analysis identified four novel putative AD-associated loci, for which corresponding p-values of the sex-specific FBATs achieve the level of 2e-07. Most notably, our analyses nominate *ZBTB7C* to represent a sex-specific AD gene. *ZBTB7C* encodes the Zinc Finger and BTB Domain Containing 7C, a transcriptional repressor of membrane metalloproteases (MMP). Members of this MMP family have been implicated in AD neuropathology in previous work^31^.

## Results

We used a combined dataset from two WGS family-based cohorts: The NIMH Alzheimer’s disease genetics initiative study (NIMH)^32^ and the family component of the NIA ADSP sample(NIA)^33^. To reduce the number of misclassified unaffected individuals, we performed a case-only analysis and focused on strong sex-specific effects. Our combined dataset contained 18,413,698 variants, after performing regular quality control and filtering by genotyping rate (Methods), in 2,247 individuals from 605 families (Table 1).

**Table 1:** Subject characteristics.

Cohort	Type	Number of families	Self-reported ancestry (n european/n african and african-american/n other)	n females/n males	n total (n cases)	Mean (SD) age at onset in cases	Mean (SD) age at onset in male cases	Mean (SD) age at onset in female cases
NIMH families	Family-based	446	1328/54/11	948/445	1393 (966)	71.9(8.45)	70.2(9.17)	72.5(8.07)
NIA ADSP families	Family-based	159	515/45/294	539/315	854 (543)	73.5(9.15)	72.1(9.27)	74.4(8.99)

SD – standard deviation.

### Sex-specific only and joint FBAT analysis

**Figure 1:**
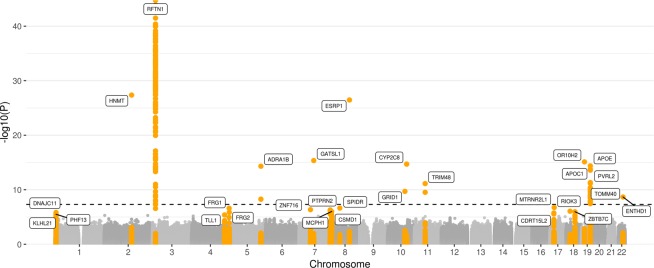
Manhattan plot for the joint FBAT-GEE analysis. The dashed line corresponds to the significance level p < 5e-08. Highlighted are genes, which correspond to loci with p < 5e-06.

**Figure 2:**
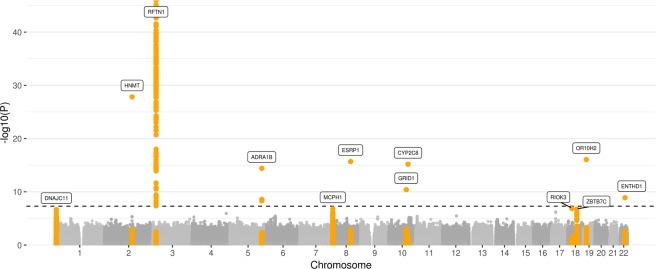
Manhattan plot for the FBAT sex-specific only analysis. The dashed line corresponds to the significance level p < 5e-08. Highlighted are genes, which correspond to loci with p < 5e-06.

In our primary analysis, we tested for a joint signal of main genetic effect for AD affection status and sex-specific interaction effect. Many of the identified genome-wide significant variants were located in the *APOE* gene cluster, clearly driven by the main, i.e. not sex-specific, effect (Fig. 1 and Supplementary Fig. 1, Methods). To disentangle the main effects from the sex-specific effects, we performed a sex-specific AD analysis (Fig. 2 and Supplementary Fig. 2, Methods). To this end, we excluded 2,864,446 variants, some of which showed genome-wide significant association, because they were located in loci, which were found to have a pseudogene on either X or Y chromosome (for example *RFTN1*) and/or did not pass quality control in TOPMED (Methods, Supplementary Figs. 2 and 3).

After this additional QC filtering, described in the previous paragraph and Methods, and after excluding the markers eliciting significant associations in the *APOE* region we found 16 variants with p_joint_ <= 5-06 (Supplementary Table 1). Among these, rs1008912 (*GRID1*; Glutamate Ionotropic Receptor Delta Type Subunit 1, chromosome 10) had p_joint_ = 2.01e-10 and p_inter_ = 3.97e-11 with an over-transmission of the minor allele to affected males. Additionally, rs181239893 (*RIOK3*; RIO Kinase 3, chromosome 18) had p_joint_ = 8.78e-07 and p_inter_ = 1.42e-07 with an over-transmission of the minor allele in males and rs13259125 (MCPH1, Microcephalin 1, chromosome 8) had p_joint_ = 9.84e-07 and p_inter_ = 1.5e-07 with an over-transmission of the minor allele in affected males. Finally, rs1944572 (*ZBTB7C*; Zinc Finger And BTB Domain Containing 7C, chromosome 18) had p_joint_ = 1.09e-06 and p_inter_ = 1.83e-07 with an over-transmission of the minor allele to affected females. (Table 2).

**Table 2:** Association statistics for top sex-specific AD associated variants with p_joint_ <= 5e-06 and p_inter_ <= 5e-07.

Chro-mo-some	Position (GRCh37)	Rs ID	A1 (effect)	A2 (other)	Effect allele fre-quency (NIMH + NIA)	Effect allele fre-quency (NIMH only)	Effect allele fre-quency (NIA only)	Nearest gene	Qua-lity in TOP-Med	has pseud-ogene on X or Y	Chi-square statistic (FBAT GEE)	P-value (FBAT GEE)	Number of infor-mative families	Z score (sex-spec-ific)	P-value (sex-specific)	Effect allele fre-quency in affected males (NIMH + NIA cohort)	Effect allele fre-quency in affected females (NIMH + NIA cohort)	Effect allele fre-quency in affected males (ADNI cohort)	Effect allele fre-quency in affected females (ADNI cohort)
10	88138165	rs1008912	C	T	0.082	0.085	0.077	GRID1	PASS	FALSE	44.657	2.01E-10	108	−6.608	3.97E-11	0.133	0.059	0.046	0.048
18	21048308	rs181239893	A	C	0.006	0.009	0.001	RIOK3	PASS	FALSE	27.891	8.78E-07	20	−5.263	1.42E-07	0.021	0.000	0.002	0.005
8	5824905	rs13259125	T	C	0.301	0.306	0.293	MCPH1	PASS	FALSE	27.662	9.84E-07	207	−5.252	1.50E-07	0.340	0.284	0.303	0.330
18	45866243	rs1944572	T	C	0.378	0.363	0.404	ZBTB7C	PASS	FALSE	27.45	1.09E-06	230	5.216	1.83E-07	0.342	0.394	0.358	0.388

Allele frequencies in affected males and females are reported in the combined NIMH + NIA cohort and, additionally, in an independent ADNI WGS cohort with unrelated subjects.

We next sought to eliminate the possibility that the association signals around our top regions were affected by mismapped reads from the X or Y chromosome. We found that variants in *GRID1* and *RIOK3* are located in repeat regions within retrotransposons. In addition, a BLAST analysis of these loci identified many matching sequences among the whole genome. Thus, lack of associated variants in LD (Supplementary Figs. 4 and 5) and the fact that those two loci are located in repeat regions suggest that they could represent read errors or mismapped reads. In contrast, *ZBTB7C and MCPH1* were mapped to only one region based on the BLAST analysis. Furthermore, there were additional variants in linkage disequilibrium with rs1944572 and rs13259125 that lent further statistical support for the sex-specific association with AD (Supplementary Figs. 6 and 7). Since our combined family-based dataset consisted to a large extent of sibships without observed parents or with only one observed parent, standard Mendel QC assessment was based only on two families, where both parents were observed. We next searched for additional Mendelian inconsistencies among our top associations in nuclear families with at least one parental genotype observed (Methods). Based on 133 such nuclear families, we didn’t observe any Mendelian errors for all 4 loci.

Next, we performed a case-only FBAT association analysis in males and females separately (Methods, Supplementary Table 1). All four variants exhibited opposite effects in males and females. Specifically, the minor allele of rs1008912 (*GRID1*) was genome-wide significant for protection in females (p = 1.83e-10) and significant for risk in males (p = 3.75e-07). Rs181239893 (*RIOK3*) and rs13259125 (MCPH1) showed the same pattern: protection in females and risk in males (Supplementary Table 1). However, the minor allele of rs1944572 (*ZBTB7C*) exhibited an opposite direction: risk in females (p = 1.28e-06) and protection in males (p = 8e-05). Together, these data illustrate AD-associated variants with opposite effects between males and females.

Finally, we performed a robust gene-environment FBAT interaction test by Hoffman *et al*.^23^ for the top selected variants, in which the environmental variable was selected to be sex. The results (Table 3) of this test confirmed the sex-specific signals and the validity of our approach. The p-values of this approach are slightly less significant than for the original analysis, as the approach by Hoffmann *et al*. has additional robustness features.

**Table 3:** Results of gene-environment FBAT interaction test from Hoffmann et al. for top 4 variants.

Strategy	Model	RS ID	Trait	Environment	Number of informative families	P-value
Hybrid	additive	rs1008912	Affection.Status	Sex	111	7.53E-06
Hybrid	additive	rs181239893	Affection.Status	Sex	18	0.00038
Hybrid	additive	rs13259125	Affection.Status	Sex	221	3.35E-06
Hybrid	additive	rs1944572	Affection.Status	Sex	252	6.57E-05

### Sex-specific effects in known AD genes

Next, we sought to identify sex-specific effects in known AD susceptibility loci and gained access to summary statistics from two large AD GWAS with approximately 455,000 individuals and approximately 64,000 individuals^20,21^. In Supplementary Tables 2 and 3 we list the reported genome-wide significant variants and our corresponding sex-specific results for these variants in our WGS dataset. Three loci, *BIN1* (rs4663105 in Jansen *et al*.; rs6733839 in Kunkle *et al*.) with elevated risk in females, *KAT8* (rs59735493 in Jansen *et al*.) with elevated risk in males and *FERMT2* (rs17125924 in Kunkle *et al*.) with elevated risk in males, showed nominally significant (p_inter_ < 0.05) sex-specific AD association. We further report, based on our WGS dataset, all nominally significant variants (p_inter_ <= 0.05) for sex-specific AD effects 500 kb up- and downstream of the reported variants from those two studies (Supplementary Table 4). The top loci included *CASS4* (p_inter_ = 2.2e-05) with elevated risk for males and protection for females, and *KLK5* (p_inter_ = 9.6e-05) with elevated risk for females and protection for males.

## Discussion

Genetic associations may have sex differences for complex human diseases. We performed a whole genome sequencing study of sex-specific effects in AD. To our knowledge, this is the first large family-based WGS study for sex-specific AD effects to date. We identified four loci that exhibited sex-specific association with AD. However, at the quality control filtering stage, we found two of the four loci are located within transposable elements of the human genome. Transposable elements make up almost half of the whole genome and they have recently been shown to be important for disease heritability^34^. But the lack of other variants in linkage disequilibrium with those two loci showing association with AD, raises questions about their validity as novel sex-specific AD loci and suggests that *GRID1* and *RIOK3* are likely artifacts, emphasizing the importance of standardized additional critical quality assessment of sequencing data when performing WGS sex-specific or other stratified analyses. Meanwhile, for the third and fourth loci at *MCPH1* and *ZBTB7C*, BLAST analysis revealed only one match on the correct chromosome, and additional variants in linkage disequilibrium with the *ZBTB7C* SNP, rs1944572, and the *MCPH1* SNP, rs13259125, also exhibited sex-specific association with AD. These data support *ZBTB7C* and *MCPH1* as a novel AD genes in which the minor allele of rs1944572 conferred risk for AD in females and protection in males and rs13259125 conferred risk for AD in males and protection in females.

Although many consortia have combined their efforts in collecting WGS data, currently, few WGS datasets are publicly available. For our top signals, we sought replication in the ADNI cohort, for which WGS data is available on 494 affected individuals. A proper assessment using the same methodology was impossible in this dataset, due to different study design, different available loci for analysis and small sample size, deeming the study not reliable for replication. But we note, that, for *ZBTB7C* we observed similar minor allele frequency (MAF) differences between affected males and females as in our family-based cohorts, although the sample size was too small to obtain significance. We also note that for *MCPH1* we observed a higher MAF frequency in females, than in males, as opposed to our study, which is not supportive of our finding.

*MCPH1*, Microcephalin 1, encodes a response protein for DNA damage, which is also implicated in chromosome condensation. The encoded protein may play a role in regulating the development of cerebral cortex in the fetal brain and neurogenesis^35–37^. Common variants in the *MCPH1* locus were shown to be associated with brain structure measures, such as brain volume and cortical area^38^. However, the same study could not replicate these findings for *MCPH1* in the ADNI dataset. Another study examined AD association with four microcephaly genes, including *MCPH1*^39^ and did not find convincing evidence, that *MCPH1* is associated with AD.

Our most robust finding was for sex-specific association with *ZBTB7C*, demonstrating a protective effect in males (and risk in females). This gene encodes Zinc Finger and BTB Domain Containing 7C, a transcription factor, which is expressed in the brain (GTEX) and is a known repressor of membrane metalloproteases (MMP 8,10,13,16)^40^. Recently, a study by Blue *et al*. identified variants associated with age at onset of AD in *ZBTB4*, another gene from the same gene family, located on chromosome 17^41^. In addition, knock out of *ZBTB7C* in mice led to decreased glucose blood levels^42^. Moreover, this same study showed that Zbtb7c deacetylated forkhead box O1 (Foxo1), leading to increased Foxo1 binding and transcriptional activation of genes involved with glucogenesis. Interestingly, the FOXO families of transcription factors have previously been implicated in AD pathogenesis by influencing neuronal survival^43^ and Aβ-induced neuroinflammation^44^. *ZBTB7C* has also been suggested as a susceptibility gene for ischemic stroke through modulation of neuronal apoptosis^45^. Finally, a paternally inherited translocation of *ZBTB7C* has been associated with non-syndromal mental retardation in male twins^46^. In a search for other variants in linkage disequilibrium with the sex-specific, AD-associated SNP in *ZBTB7C*, rs1944572, one rare (minor allele frequency = 0.008 in gnomAD) missense variant, rs61729532 (Proline250Serine) was detected, which was in strong linkage disequilibrium with rs1944572 (D’ = 1), and yielded a p_inter_ = 0.028 for sex-specific association with AD with an elevated risk for females and protection for males. Future studies assessing how this missense mutation affects *ZBTB7C* function, particularly in female versus males brain tissue, would be warranted.

Our study has several limitations. First, as noted in the methods, the test we use is particularly powerful in scenarios where the sex-specific effects are in opposite directions, but might miss same direction sex-specific effects of different magnitude. However, it is worth noting that separate tests in males and females are not sufficient to prove interaction, even if both are statistically significant and effect estimates point in opposite directions. Larger sample sizes and more sensitive approaches are needed to detect same direction sex-specific effects. Collection of large datasets with suitable families requires more time and resources as compared to population-based or case-control cohorts. Next, our study was dominated by samples of European ancestry. Racial and ethnic differences in AD prevalence and genetics are recognized in the literature^1,47–49^. Although FBAT is robust to confounding due to population structure, an expanded set of underrepresented populations is necessary to identify AD sex-specific effects unique to other populations.

In summary, we have developed and employed an FBAT-approach using an affected-only analysis to detect sex-specific AD effects. During the analysis, we encountered the need for additional sex-specific filtering steps that have not previously been considered for the association analysis of a WGS scan to reduce the number of false positive findings. Using our analysis approach, we initially identified four loci showing sex-specific association with AD risk. However, after additional quality control filtering, the only candidate remaining was a variant in *ZBTB7C*, rs1944572, which conferred increased risk for AD in females and protection from AD in males and showed similar MAF differences between affected males and females in an independent cohort. Recently, methods have started to emerge, that address sex chromosome related mismapping in WGS data, but these approaches are still early in development^29^. Based on our experience, we recommend careful re-mapping of all autosomal variants to search for potential homology on the sex-chromosomes which may drive spurious associations. To the best of our knowledge, our study represents the first large family-based WGS analysis for sex-specific associations with AD. Similar analyses of additional AD WGS samples are needed to confirm our putative association with *ZBTB7C*, to identify additional novel sex-specific AD loci, and to better understand sex-specific genetic features and potential pathways for AD development.

## Methods

### Cohort description and sequencing

Briefly, sequencing in NIMH was performed by Illumina HiSeq 2000. Alignment to the human reference genome (GRh37) was done with bwa-mem^50^ (v0.7.7, default parameters). Variants were jointly called for each family using FreeBayes^51^ (v0.9.9.2-18) and GATK^52^ (v3.0) best practices method as part of the bcbio-nextgen workflow^53^ before being squared-off with bcbio.recall across the whole cohort to distinguish reference calls from no variant calls. Library and read quality were assessed using FastQC^54^ (v0.10.1) and Qualimap^55^ (v0.7.1). Variant calls in vcf format for the families from the NIA ADSP cohort were obtained from the National Institute on Aging Genetics of Alzheimer’s Disease Data Storage Site (NIAGADS) under accession number: NG00067 and the database of Genotypes and Phenotypes (dbGaP) under accession number: phs000572v8p4. Both cohorts: NIMH^32^ and the family component of the NIA ADSP sample^33^ consisted of multiplex AD families with affected and unaffected siblings (Table 1). A subject was considered to be affected, if he/she was included in one of the following categories: “Definite AD”, “Probable AD” or “Possible AD”. Unaffected subjects had either no dementia, suspected dementia (34 subjects) or non-AD dementia (4 subjects). It is important to note that NIA ADSP families by design did not include individuals with two APOE-ε4 alleles. After standard quality control both cohorts were merged together as described in the next section.

The Alzheimer’s Disease Neuroimaging Initiative (ADNI) was used to lookup minor allele frequencies in males and females for the top candidate findings. Data was obtained from the Alzheimer’s Disease Neuroimaging Initiative (ADNI) database (adni.loni.usc.edu). The ADNI was launched in 2003 as a public-private partnership, led by Principal Investigator Michael W. Weiner, MD. The primary goal of ADNI has been to test whether serial magnetic resonance imaging (MRI), positron emission tomography (PET), other biological markers, and clinical and neuropsychological assessment can be combined to measure the progression of mild cognitive impairment (MCI) and early Alzheimer’s disease (AD).

### Regular quality control

We used PLINK^56,57^ v1.9 to calculate most of the quality metrics. Initially, we had 1432 individuals (affected/unaffected siblings) from the NIMH cohort and 873 individuals from the NIA. Nineteen individuals in NIA were removed because they were marked as either replicates, duplicates or had a bad GWAS concordance. Three individuals in NIMH were removed because they were outliers based on genotyping rate and inbreeding coefficient. Based on estimated identity by descent (IBD) sharing coefficients we identified 12 duplicate pairs and 24 individuals with wrong family assignments in NIMH. After filtering the analyzed dataset was composed of two WGS familial AD cohorts with 1,393 individuals (NIMH; 446 families) and 854 individuals (NIA; 159 families), which were merged together. For variant quality control we performed the following: we have used only variants which passed all quality control filters in the vcf file (marked by “PASS”), excluded multiallelic variants, monomorphic variants, singletons (i.e. variants with only one alternative allele across the dataset), indels and variants which had one missing allele among 2 alleles in an individual. The remaining variants were filtered based on Mendel errors and genotyping rate (95%).

### Additional quality control

We performed additional quality control after our main analysis to eliminate additional sequencing and calling errors. We excluded variants, which were not called in TOPMed^58,59^ – a large WGS database with >100,000 individuals sequenced jointly. We also screened a pseudogene database^60^ for genes, which had known pseudogenes on either X or Y chromosome. This allowed us to eliminate possible mapping errors.

For the four novel candidate SNPs, we performed an additional, non-standard Mendel error check. We utilized the genetic data for 133 nuclear families where one parental genotype is available and screened for Mendelian inconsistencies (discordant homozygous genotypes between parent and offspring). No Mendelian inconsistency was found.

In order to eliminate possible mismapping issues we performed a local alignment (BLAST) for the four candidate loci using the BLAT tool from Ensembl Genome Browser^61^. As the query we used a flanking sequence centered around the variant of interest. The query length was 201 basepairs, 100 basepairs from each side of the selected variant.

### Sex-specific FBAT analysis

The NIMH and NIA samples are studies of extended families of multiple generations with affected and unaffected siblings. While the genotypes of all offsprings regardless their phenotypes are observed, most of the parents are missing. As the ascertainment condition for both samples was that at least one of the offspring has late onset AD, we decided to perform a case-only analysis, minimizing the number of misclassified unaffected individuals who might have developed the disease later in life. In order to maximize the power of the FBAT-statistic, we set the phenotype of the unaffected offspring as missing, so that their genotype information can still be used in the construction of the sufficient statistics for each family.

We used the FBAT software^62^ and the following framework to perform a sex-specific family-based association study for Alzheimer’s disease. Briefly, The FBAT score statistic can be described as:

$$U={\sum }_{i=1..nj=1..{n}_{i}}{T}_{ij}({X}_{ij}-E({X}_{ij}|{S}_{i})),$$

where n is the number of families, n_j_ is the number of offsprings in the family i, X_ij_ is the genotype, S_i_ – family sufficient statistic for parental genotypes, T_ij_ – the coded trait of offspring. Usually T_ij_ is the phenotypic residual and corresponds to *T*_*ij*_ = *Y*_*ij*_ − *μ*, where Y_ij_ is the phenotype of the offspring and µ is the offset parameter, which we set to 0.15 to approximately correspond to the population prevalence of Alzheimer’s disease. Since we use only affected offsprings in our analysis, we modify T_ij_ to incorporate sex-specific effects into the test statistic and define it as following: *T*_*ij*_ = (*Y*_*ij*_ − *μ*)(*Z*_*ij*_ − 0.5), where (*Y*_*ij*_ − *μ*) is constant, Zij is 0, if the offspring is male and 1 if it is female. Our modified FBAT score statistics is:

$$U={\sum }_{i=1..nj=1..{n}_{i}}({Y}_{ij}-\mu )({Z}_{ij}-0.5)({X}_{ij}-E({X}_{ij}|{S}_{i})).$$

It is important to note that this coding of the sex-specific effect will be especially efficient if the genetic effect direction is different between sexes.

In order to test the joint effect we used FBAT-GEE^63^ on two phenotypes: *T*_1_ = *Y* − *μ* and *T*_2_ = (*Y* − *μ*)(*Z* − 0.5), which were constructed as described above.

Additionally, we used a robust gene by environment test, developed by Hoffmann *et al*.^23^. For this we used the function “fbatge” from the “fbati” package. We used sex as the environmental variable and utilized the hybrid test strategy, which is more efficient for sibships without parental genotypes.

R^64^ v3.5.0 and Locuszoom^65^ were used to generate all plots.

### Signal direction assessment

In order to identify the AD risk direction for our sex-specific analysis, we performed a case-only family-based analysis for AD affection status with FBAT in males and females separately. We kept all the genotypes to calculate sufficient statistics for each family. By setting the phenotype of one of the sexes to missing, we were able to observe the effect direction for the tested allele in males and females.

### Assessment of known AD susceptibility regions

We used summary statistics from two recent large genome-wide association studies of AD^20,21^. We sought to identify whether any of the reported genome-wide significant loci from those studies showed a sex-specific effect in our analysis. Additionally, we extended our lookup to all variants, located 500 kb up- and downstream of the reported variants in the papers.

### Ethical statement

This research project is approved by the Institutional Review Board (IRB) (2015P000111) at Massachusetts General Hospital. Informed consent was obtained from all subjects. All methods were carried out in accordance with relevant guidelines and regulations.

## Supplementary information

Supplementary information.

Supplementary tables 1-3.

Supplementary table 4.

## Data Availability

The NIMH dataset analysed during the current study is available from the corresponding author on reasonable request. The family component of the NIA ADSP WGS dataset is available from DSS NIAGADS under accession number: NG00067. The ADNI WGS dataset is available at http://adni.loni.usc.edu/.
